# Evolutionarily significant A-to-I RNA editing events originated through G-to-A mutations in primates

**DOI:** 10.1186/s13059-019-1638-y

**Published:** 2019-02-04

**Authors:** Ni A. An, Wanqiu Ding, Xin-Zhuang Yang, Jiguang Peng, Bin Z. He, Qing Sunny Shen, Fujian Lu, Aibin He, Yong E. Zhang, Bertrand Chin-Ming Tan, Jia-Yu Chen, Chuan-Yun Li

**Affiliations:** 1 Laboratory of Bioinformatics and Genomic Medicine, Beijing Key Laboratory of Cardiometabolic Molecular Medicine, Institute of Molecular Medicine, Peking University, Beijing, 100871 China; 2 Department of Central Research Laboratory, Peking Union Medical College Hospital, Peking Union Medical College and Chinese Academy of Medical Sciences, Beijing, China; 3 Biology Department, University of Iowa, Iowa City, IA USA; 4 State Key Laboratory of Integrated Management of Pest Insects and Rodents & Key Laboratory of the Zoological Systematics and Evolution, Institute of Zoology, Chinese Academy of Sciences, Beijing, China; 5 Department of Biomedical Sciences and Graduate Institute of Biomedical Sciences College of Medicine, Chang Gung University, Tao-Yuan, Taiwan; 6 Molecular Medicine Research Center, Chang Gung University, Tao-Yuan, Taiwan; 7 Department of Cellular and Molecular Medicine, University of California, San Diego, La Jolla, CA 92093-0651 USA

**Keywords:** RNA editing, Origination, Primate evolution, G-to-A mutation, Population genetics

## Abstract

**Background:**

Recent studies have revealed thousands of A-to-I RNA editing events in primates, but the origination and general functions of these events are not well addressed.

**Results:**

Here, we perform a comparative editome study in human and rhesus macaque and uncover a substantial proportion of macaque A-to-I editing sites that are genomically polymorphic in some animals or encoded as non-editable nucleotides in human. The occurrence of these recent gain and loss of RNA editing through DNA point mutation is significantly more prevalent than that expected for the nearby regions. Ancestral state analyses further demonstrate that an increase in recent gain of editing events contribute to the over-representation, with G-to-A mutation site as a favorable location for the origination of robust A-to-I editing events. Population genetics analyses of the focal editing sites further reveal that a portion of these young editing events are evolutionarily significant, indicating general functional relevance for at least a fraction of these sites.

**Conclusions:**

Overall, we report a list of A-to-I editing events that recently originated through G-to-A mutations in primates, representing a valuable resource to investigate the features and evolutionary significance of A-to-I editing events at the population and species levels. The unique subset of primate editome also illuminates the general functions of RNA editing by connecting it to particular gene regulatory processes, based on the characterized outcome of a gene regulatory level in different individuals or primate species with or without these editing events.

**Electronic supplementary material:**

The online version of this article (10.1186/s13059-019-1638-y) contains supplementary material, which is available to authorized users.

## Introduction

RNA editing is a post-transcriptional mechanism that introduces differences between RNA and its corresponding DNA sequence [1]. One type of RNA editing events, the A-to-I editing, is catalyzed by adenosine deaminase acting on RNA (ADARs) acting on dsRNAs. Due to the prevalence of dsRNA structures formed by the inverted repeated *Alus* in primates, which are the preferred substrates of ADARs, A-to-I editing is the most common type of RNA editing in primates [2]. The recent next-generation sequencing technology dramatically accelerated the study of A-to-I editing regulation on a genome-wide scale [3–6], with nearly 3% of the human genome estimated to be subject to the regulation [4].

Most A-to-I RNA editing sites in primates are contributed by the expansion of primate-specific *Alu* elements [6, 7]. Of these widespread A-to-I RNA editing sites in primates, only a small proportion is located in well-recognized functional regions, such as the protein-coding or miRNA encoding loci, and presumably implicated in altering sequences of proteins or miRNAs [1, 3, 5, 8, 9]. As population genetics analyses have recently hinted at the functional relevance for editing sites in other genomic regions, functional dissection of these pervasive RNA editing sites has emerged as a critical issue in the field [5, 10–12]. While several recent studies have suggested the potential crosstalk between RNA editing and other regulatory processes, such as alternative splicing, piRNA biogenesis and cytosolic dsRNA response [10, 11, 13, 14], an in-depth functional perspective of the widespread A-to-I editing sites in primate evolution remain to be addressed.

Recently, a group of A-to-I RNA editing sites has been reported in candidate gene studies to be genomically encoded as non-editable nucleotide in other closely related species [15, 16], representing a recent birth or death process of RNA editing through DNA point mutations. Importantly, comprehensive characterization of this subset of RNA editome, if exists, could advance the evolutionary and functional interrogation of primate RNA editing regulation in the following regards. First, because RNA editing identification depends heavily on the quality and sequencing depth of the transcriptome, defining a species-specific editing site in a comparative transcriptome study could be confounded by multiple factors such as technical limitation in ascertaining true absence of editing from the failure of detection [17]. In contrast, a distinctive list of RNA editing gain or loss events through DNA point mutations constitutes a valuable alternative to confidently define newly originated, species-specific RNA editing events. This possibility is supported by the notions that the editing regulation in out-group species is explicitly absent (genomically encoded as non-editable nucleotides) and that the ancestral state of these sites could be inferred with sequence data of multiple reference species. This unique group of RNA editing events with evolutionary age may thus provide a basis for studying the evolutionary significance of RNA editing in primate evolution. Second, comparative genomics analyses of these RNA editing events could also provide functional connection of RNA editing to particular gene regulatory processes. As the editing sites detected in one species were genomically encoded as non-editable nucleotides in the other species, a cross-species comparison of the outcome of a gene regulatory level may provide clues to the functional implications of these species-specific editing sites, which would further illuminate the general functions of RNA editing regulation.

Although cases of the birth or death process of RNA editing through DNA point mutation have been reported, the generality of this phenomenon on a genome-wide scale, the models underpinning the phenomenon, and the applications of these events in evolutionary and functional interrogation of RNA editing regulation remain largely unresolved. In particular, this phenomenon on the population level, in which the A-to-I RNA editing sites detected in some individuals are genomically encoded as non-editable nucleotides in other individuals, would complement these analyses. However, this type of polymorphic editing sites is intentionally omitted primarily due to the potential false positives of these events and their consequent removal by the editing-calling computational pipelines [4, 18–20].

## Results

### Recent gain and loss of *Alu*-associated A-to-I RNA editing through DNA mutation in rhesus macaque

**Fig. 1:**
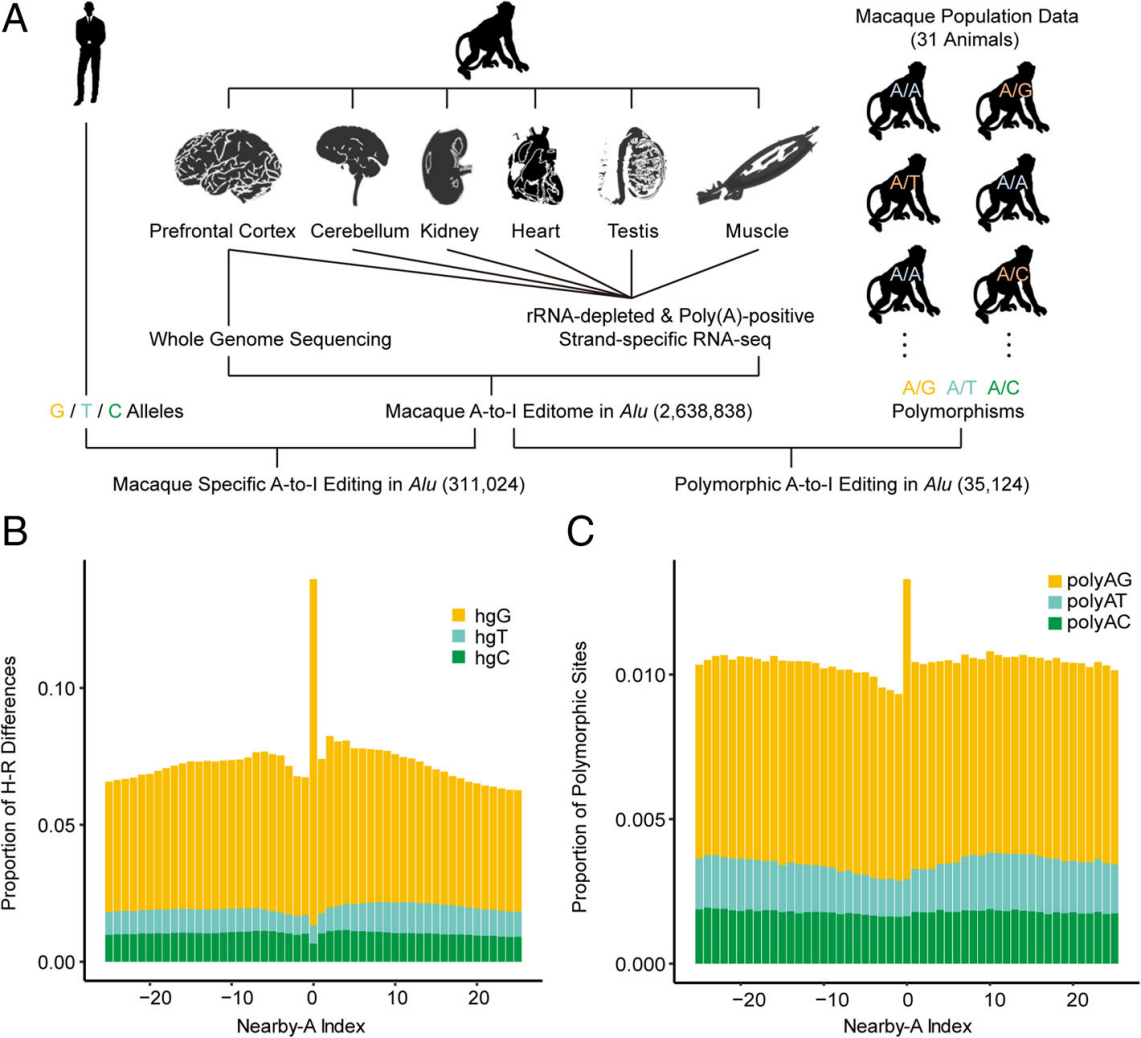
Genome-wide identification of the species-specific and polymorphic *Alu*-A-to-I RNA editing sites in rhesus macaque. **a** An overview of the experimental design. **b** The distribution of the proportions of human-macaque sequence differences is shown for the focal RNA editing sites (Index 0) as well as the nearby non-edited, homozygous A sites within 25 bp upstream or downstream of the focal editing sites (Nearby-A Index). hgG, hgT, and hgC: the macaque editing sites genomically encoded as G, T, and C in human orthologous sites, respectively. **c** The distribution of the proportions of polymorphic sites within macaque population is shown for the focal editing sites (Index 0) and the nearby homozygous A sites within 25 bp upstream or downstream of the focal editing sites (Nearby-A Index). polyAG, polyAT, and polyAC: the macaque editing sites located on the A/G, A/T and A/C polymorphic sites in the macaque population, respectively

To study the recent gain and loss of RNA editing in primates, we used rhesus macaque as a model animal and profiled the RNA editome in six tissues (prefrontal cortex, cerebellum, heart, kidney, muscle, and testis) of the same macaque animal (Fig. 1a). To this end, we applied a stringent RNA editing-calling pipeline on the poly(A)-positive and rRNA-depleted RNA-seq data (Additional file 1: Table S1), which was implemented based on our previous experiences in technical accuracy [5, 10] (“Methods”). Among the 2,828,972 candidate sites identified, 2,735,258 sites (or 96.69%) were located on the *Alu* repeat elements. For these *Alu*-associated candidates, 2,638,838 sites (or 96.47%) were associated with A-to-I transitions, which were used in the following analyses (Additional file 2: Table S2). Notably, these *Alu*-associated A-to-I RNA editing sites verified multiple known features of RNA editing events in primates, such as a conserved sequence motif for ADARs recognition nearby the editing sites, as well as a quantitative correspondence between the tissue-specific profile of the RNA editome and the expression level of *ADARs* in that tissue (Additional file 2: Figure S1A, S1B), indicating that these candidate sites represent bona fide RNA editing events mediated by ADARs.

As it has been reported in some case studies that A-to-I RNA editing sites could be genomically encoded as non-editable nucleotide in other species, representing a recent gain and loss process of RNA editing through DNA mutations [15, 16], we first examined the generality of such a phenomenon across species, by performing a genome-wide comparative analysis between human and rhesus macaque. Notably, for the 2,223,166 *Alu*-associated A-to-I RNA editing sites detected in the macaque animal with correspondence to the human orthologous sites, 281,578 (12.67%) are genomically encoded as G in human on the basis of the reference genome, with 255,056 (11.47%) sites (hgG) being fixed in the human population on the basis of the 1000 Genomes Project [21]—a significantly higher proportion than the nearby non-edited, homozygous A sites (Nearby-A, Fisher’s exact test, *P* value < 2.2 × 10^− 16^, Fig. 1b). Even when controlled for the variation of position-dependent mutation rates, by requiring the control sites located in the same *Alu* with the same trinucleotide context, the editing sites still showed a significantly higher level of overlap with A/G divergent sites between human and rhesus macaque (Fisher’s exact test, *P* value < 2.2 × 10^− 16^, Additional file 2: Figure S2A). In contrast, 29,446 (1.32%) sites were genomically encoded in human as Cs or Ts, with 27,212 (1.22%) fixed, a proportion not significantly higher than that of the nearby non-edited, homozygous A sites as a background (Fisher’s exact test, *P* value = 1, Fig. 1b).

We then set out to investigate whether this phenomenon exists within macaque population. Existing computational pipelines are conventionally designed to remove candidate editing sites located on previously annotated bi-allelic polymorphic sites [18, 22], as these sites may represent heterozygous sites that are wrongly assigned as homozygous A sites due to biased representation of two alleles in the genome sequencing with inadequate sequencing depth. In this study, given that the genome of the macaque animal used to identify RNA editing sites was sequenced with high coverage, we were able to distinguish these two types of sequence variations with high confidence. By combining the RNA editing profile and the macaque polymorphism map as we previously defined with a population of 31 macaque animals [23] (“Methods”), we identified 27,412 A-to-I editing sites (1.04%) located on the A/G polymorphic sites in the macaque population (polyAG; Additional file 3: Table S3), a percentage significantly higher than the nearby non-edited, homozygous A sites as a background (Nearby-A, Fisher’s exact test, *P* value < 2.2 × 10^− 16^, Fig. 1c). When we controlled for the variation of position-dependent mutation rates, by requiring the control sites located in the same *Alu* with the same trinucleotide context, the editing sites still showed a significantly higher level of overlap with A/G polymorphic sites in the macaque population (Fisher’s exact test, *P* value < 2.2 × 10^− 16^, Additional file 2: Figure S2B). In contrast, 7712 A-to-I editing sites (0.29%) located on the A/C or A/T polymorphic sites (polyAC or polyAT), a proportion not significantly higher than that of the background (Fisher’s exact test, *P* value = 1, Fig. 1c). The remaining 2,603,710 (98.67%) sites showed no sequence variation in the 31 macaque animals sampled in our study.

**Fig. 2:**
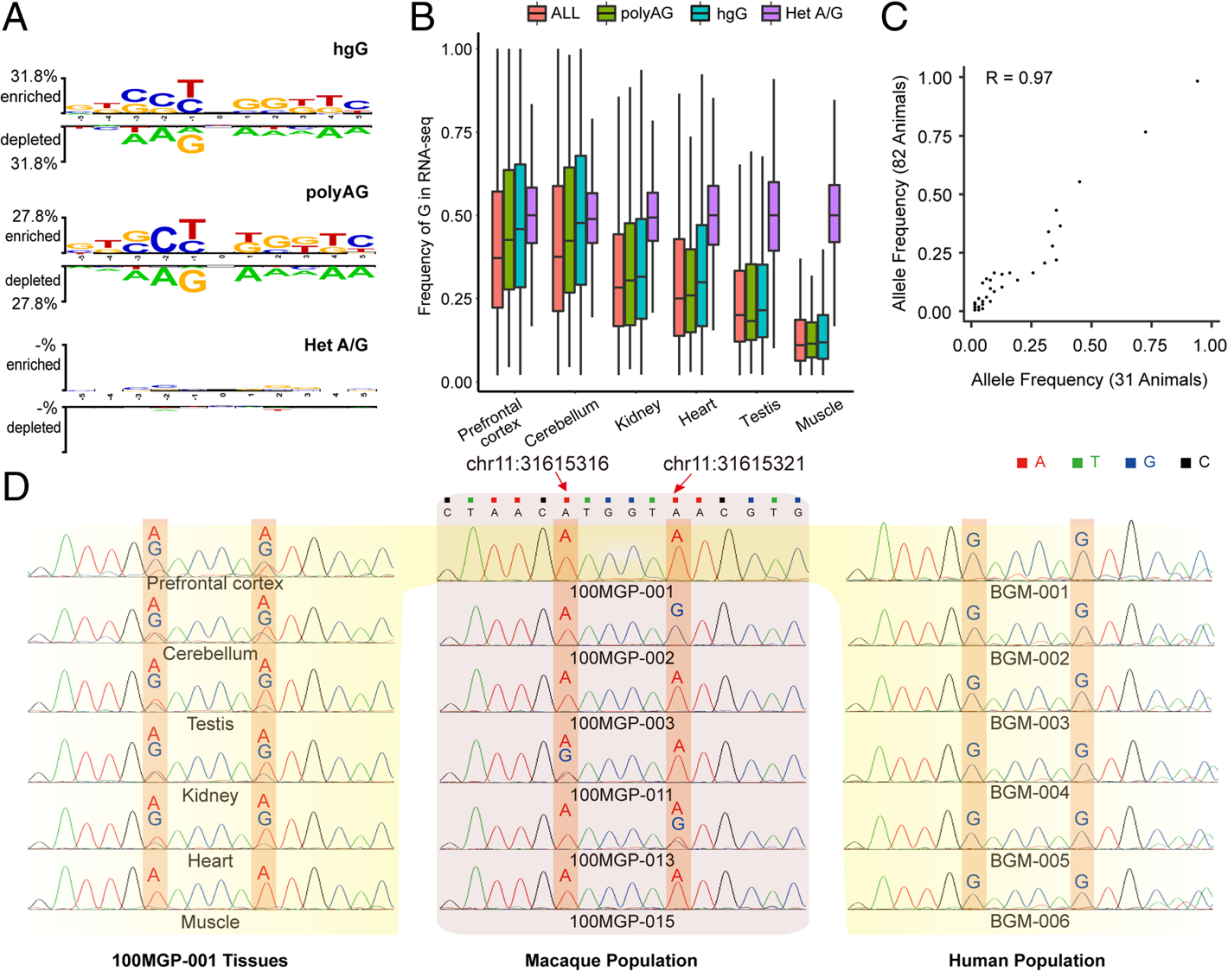
Evaluation and validation of hgG and polyAG editing sites in rhesus macaque. **a** The enriched and depleted nucleotide sequences flanking the *Alu*-associated A-to-I RNA editing sites are shown for macaque-specific editing sites (hgG), A/G polymorphic editing sites (polyAG), and A/G heterozygote sites (Het A/G) as negative control, with the level of preference or depletion presented in height proportional to the scale. **b** The distributions of editing levels in six macaque tissues, estimated as the frequency of G-harboring reads in rRNA-depleted RNA-seq data, are shown for all *Alu*-associated A-to-I editing sites (All), macaque-specific editing sites (hgG) and A/G polymorphic editing sites (polyAG). As a negative control, the frequencies of G for A/G heterozygote sites were also estimated with RNA-seq data (Het A/G). **c** Scatter plot shows the frequency of the G allele in 54 randomly selected polyAG sites, as estimated by using 31 macaque animals with whole genome sequencing, and 82 animals with targeted DNA sequencing. **d** Two exemplary polyAG/hgG editing sites located on the A/G polymorphic sites in the macaque population and also genomically encoded as G in human were verified with Sanger sequencing. Two editing sites (indicated by arrow) were identified in cDNA samples from six tissues of the same macaque animal (100MGP-001). The focal editing sites are A/G polymorphic in a macaque population of six animals, including 100MGP-001 (“Methods”), and are genomically encoded as G in a human population of six healthy individuals (“Methods”)

Several lines of evidence corroborated the hgG and polyAG editing sites as *bona fide* A-to-I editing sites instead of false positives caused by A/G heterozygotes: (i) the DNA coverage of these A-to-I editing sites is not lower than other editing sites, ruling out the possibility that these sites were wrongly assigned as homozygotes due to biased representation under lower DNA sequencing coverage (Additional file 2: Figure S1C); (ii) the local sequence context of these sites, but not for that of the A/G heterozygotes, was in well accordance with the motif of editing sites (Fig. 2a); (iii) furthermore, when estimating the frequency of G allele on mRNA level from rRNA-depleted RNA-seq data, we found that the distribution of the editing frequency of these sites is similar to the other classes of editing sites, and the pattern for editing frequency across different tissues verified the tissue distribution of *ADARs* expression [5] (Fig. 2b; Additional file 2: Figure S1B). We further experimentally verified that the polymorphic sites associated with the polyAG editing sites were *bona fide* in the macaque population. To this end, we performed targeted region re-sequencing in a larger population of 82 macaque animals, in addition to the initial macaque population of 31 animals [23]. DNA oligonucleotides were designed to target 54 randomly selected polyAG sites, followed by high-throughput sequencing of the targeted regions (“Methods”; Additional file 4: Table S4). In the new macaque population, 68.5% (37 of 54) of the polyAG sites were verified to be A/G polymorphic, and the frequencies of the G allele estimated from these two populations were highly correlated (Pearson correlation coefficient = 0.97, Fig. 2c). As for the other 17 sites undetectable as polymorphic in the new population, the frequency of the polymorphism detected in the initial population is relatively low (median = 0.03). In addition, seven randomly selected polyAG sites were also experimentally verified by PCR amplification and Sanger sequencing, and all of these sites were confirmed to be A/G polymorphic within the population (Fig. 2d; Additional file 2: Figure S1D; “Methods”). These results confirmed the authenticity of A/G polymorphic sites, and the extent of accuracy for the allele frequencies estimated with the initial macaque population.

### An excess of robust A-to-I editing events originated following G-to-A mutation

Consistent with the over-representation of A/G divergent and polymorphic sites at RNA editing sites, more transcribed hgG (6.30%) or polyAG sites (4.19%) located on *Alu* elements could be detected as editing sites in contrast to the normal adenosine sites as the background (hgA sites, 2.65%). These findings thus suggest that sites with A/G mutation might be the hotspot for the recent gain or loss of robust A-to-I editing events (Fig. 1b, c). Such a phenomenon may either represent a recent gain of RNA editing event following a G-to-A DNA point mutation, or the loss of an ancient RNA editing event through a human-specific or individual-specific A-to-G mutation. To further distinguish the two possibilities, we then set out to characterize the formation process of these events by performing ancestral state inferences based on multi-genome alignment with multiple primate species (“Methods”). For most of these A-to-I RNA editing sites, the ancestral states were inferred to be G or A, representing “editing gain” or “editing loss”, respectively (99.91% for hgG sites and 99.87% for polyAG sites). Specifically, 77.30% hgG editing sites (hgG-ancG) and 29.55% of polyAG editing sites (polyAG-ancG) in rhesus macaque follow an “editing gain model”, in which the allele of the human-macaque ancestor or the allele of the most recent common ancestor of macaque polymorphic sites is G. In contrast, 22.61% hgG editing sites (hgG-ancA) and 70.32% of polyAG editing sites (polyAG-ancA) follow an “editing loss model” in which the ancestor allele is A.

**Fig. 3:**
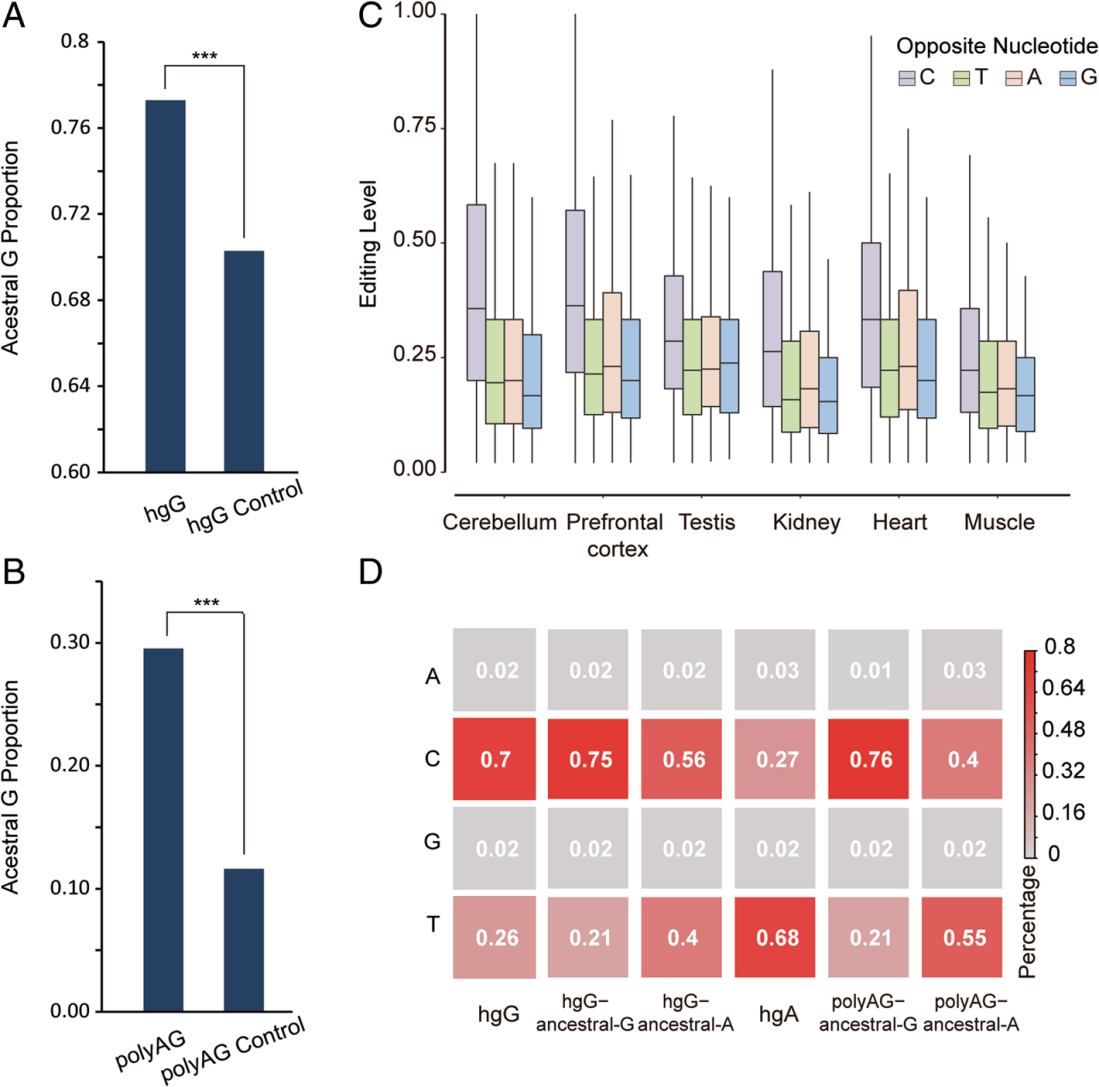
G-to-A mutation as a favorable location for the origination of robust A-to-I editing events. **a**, **b** For species-specific (hgG) and A/G polymorphic (polyAG) editing sites, the proportions of sites with ancestral states of G are shown, respectively. The proportions of the adjacent non-edited A sites fixed in the human population as G alleles (hgG Control), or non-edited, polymorphic A/G sites in macaque population (polyAG Control) are also shown as the background. **c** The distributions of the editing frequency in six macaque tissue types (prefrontal cortex, cerebellum, heart, kidney, muscle, and testis) are shown in boxplots. Sites with different opposite, base-pairing nucleotide in the mRNA secondary structure are shown separately in different bins. **d** For hgG and polyAG editing sites with ancestral states of G allele or A allele, as well as hgA editing sites as a control, the compositions of the opposite nucleotides (A, T, C, or G) are shown in heatmaps, with the levels of percentage proportional to the color scale

As hgG and polyAG editing sites showed opposite patterns for the allocation of the two models, we next sought to address possible biases introduced in the definition of these sites. In contrast to hgG sites, the definition of polyAG editing sites per se could introduce biased allocation of the two models. To this end, because the homozygous adenines in the macaque animal was the prerequisite for A-to-I editing identification, A is less likely to be the derived allele, as it is relatively difficult to identify derived, homozygous adenines in the macaque animal for A-to-I editing identification in such an occasion. The ancestral allele of these sites is thus more likely to be A, and more polyAG editing sites are thus presumably attributed to an “editing loss model”. To control for the biases, we thus introduced matched controls for the two types of sites (“Methods”). Notably, compared with the controls in adjacent non-edited A sites encoded in the human population as G nucleotides (hgG Control), or in non-edited, polymorphic A/G sites in macaque population (polyAG Control), significantly more editing sites follow the “editing gain model” (Fisher’s exact test, *P* value < 2.2 × 10^− 16^, Fig. 3a, b) for both hgG and polyAG editing sites, evidencing an excess of origination of robust A-to-I editing events following G-to-A mutation.

Taken together, the over-representation of mutations at the RNA editing sites is largely contributed to by A/G transitions, a proportion significantly higher than the genome-wide background or regions of *Alu* repeats. Notably, it seems the pattern of over-representations is largely contributed to by the sites with ancestral G (Additional file 2: Figure S3). Moreover, as for the finding of a higher proportion of transcribed, *Alu*-associated hgG or polyAG sites detected as editing sites, when dividing these hgG or polyAG sites into different categories according to their ancestral status and controlling for the variation of position-dependent mutation rates in the same trinucleotide context, we found that the over-representations are found specifically at hgG or polyAG sites with ancestral G, rather than with ancestral A (Additional file 2: Table S5).

### “Opposite C-ancestral G” pairing represents a favorable location for the origination of robust A-to-I editing event

This observation prompted us to investigate the mechanism underlying this over-represented A-to-I editing gain from G-to-A mutation sites. Presumably, an intuitive explanation is that A sites mutated from G are more suitable substrates for RNA editing machinery than those from C or T. Indeed, the editing levels for hgG sites are significantly higher than those for hgC or hgT sites, a finding supporting this hypothesis (Additional file 2: Figure S4). Examination of the dsRNA structure nearby the newly originated RNA editing sites further provided explanations to this phenomenon. Briefly, consistent with previous reports [24], we found that the A nucleotide with an opposite C nucleotide in the mRNA secondary structure represented a more favorable target for ADARs recognition, as evidenced by the significantly higher editing level (Wilcoxon test, *P* value < 2.2 × 10^− 16^, Fig. 3c). Notably, since *Alu* elements are highly analogous to each other and the *Alu* dsRNA structure is formed with nearly perfect base-pairing, the opposite positions of C nucleotides are more likely to be G nucleotides in the dsRNA structure. In the ancestral states, the positions opposite of C nucleotides are thus amenable to the origination of A-to-I editing events with detectable editing frequency. In this capacity, the G-to-A mutation site thus becomes a favorable location for the origination of robust A-to-I editing events.

As expected, when examining the dsRNA structure of the macaque transcript nearby these newly originated RNA editing sites, we found an “opposite C-ancestral G” pairing for the majority of these hgG/polyAG sites (Fig. 3d), and the hgG/polyAG sites with ancestral G have more opposite C compared to hgA sites, or hgG/polyAG sites with ancestral A (Fig. 3d). Moreover, when examining the secondary structure of the human homologous transcripts, assuming that the secondary structures have remained unchanged in the human lineage since it had a common ancestor with rhesus macaque, we found that 79.3% of the G sites were indeed paired with C in human transcripts. This finding thus supports the hypothesis that the majority of these hgG/polyAG sites were originated from ancestral sites of GC pairing.

Notably, two possible mechanisms may underpin the over-representation of “opposite C-ancestral G” pairing sites: First, it is possible that the diversity between *Alu*s was lower in the past than the present, raising the assumption that the ancestral dsRNA structure could be tighter than the younger one. In such an occasion, a bigger pool for “opposite C-ancestral G” pairing sites relaxing the dsRNA structures during the primate evolution (C-G pair to C-A pair) should be expected. Second, even when the “opposite C-ancestral G” pairing sites are not enriched during the primate evolution, a site with “opposite C-ancestral G” pairing may represent a favorable location for the origination of robust A-to-I editing event, leading to its detection in our analysis. To distinguish the two mechanisms, we introduced negative controls of hgG sites (regardless of the editing status) as a background, such as the *Alu*-associated, hgG sites with ancestral G (all hgG-ancG adenosines), as well as *Alu*-associated, hgG sites with ancestral A (all hgG-ancA adenosines). The fraction of opposite C is higher for hgG-ancG adenosines (56%) than hgG-ancA adenosines (28%), suggesting that the ancestral dsRNA is generally tighter than the current one. Notably, as a higher fraction of opposite C was found for hgG-ancG editing sites (75%) in comparison to the control group of all hgG-ancG adenosines (56%), it is plausible that the over-representation of “opposite C-ancestral G” pairing sites could be attributed to a bigger pool for these sites relaxing the dsRNA structures during the primate evolution, as well as the scenario that a site with “opposite C-ancestral G” pairing is a favorable location for the origination of robust A-to-I editing event.

The model proposed here lends support to the features of the over-representation detected. In addition, the list of hgG and polyAG editing sites also represents a valuable resource to investigate the features and functions of A-to-I RNA editing events at the population and species levels.

### A portion of the newly originated RNA editing events is evolutionarily significant

**Fig. 4:**
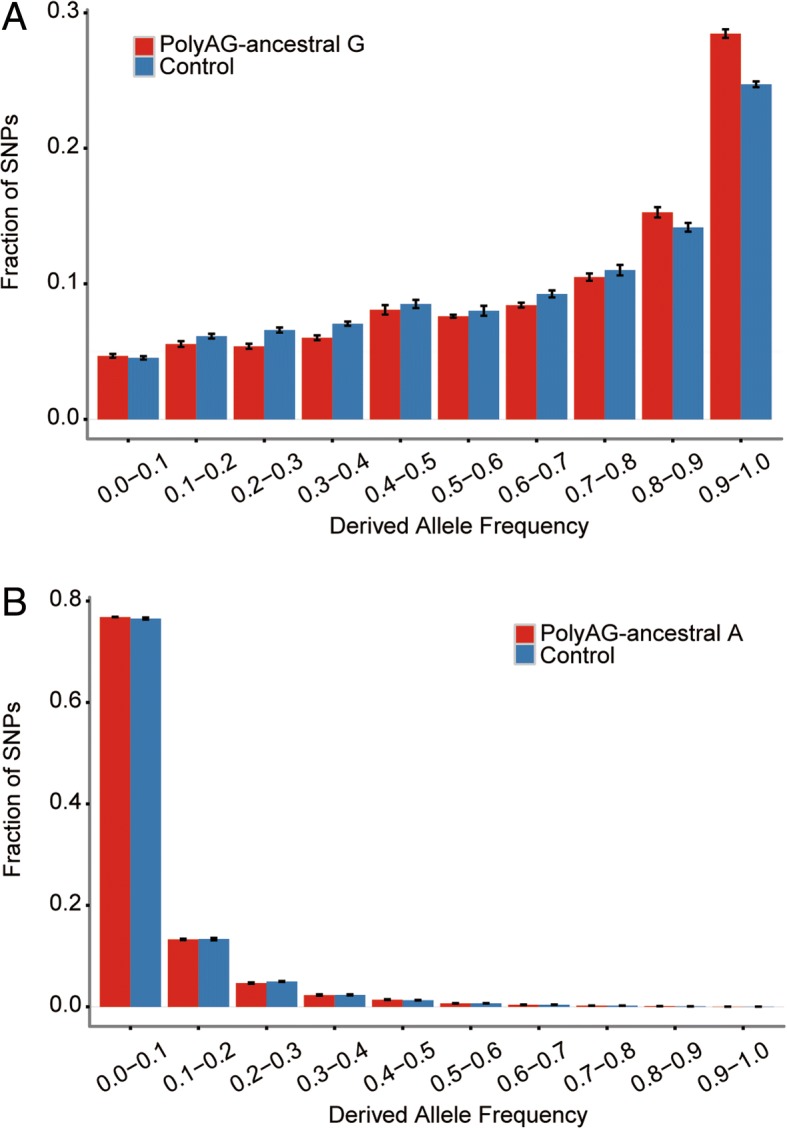
The newly originated RNA editing events are selectively constrained in general. **a** For the newly originated polyAG editing sites with ancestral state of G, a site frequency spectrum for the derived A allele is shown (Editing Gain). As a background, a list of non-editing, homozygous A sites with ancestral state of G (Control) was used to generate a site frequency spectrum for derived A allele (“Methods”). **b** For the newly originated polyAG editing sites with ancestral state of A, a site frequency spectrum for the derived G allele is shown (Editing Loss). As a background, a list of non-editing, homozygous G sites with ancestral state of A (Control) was used to generate a site frequency spectrum for derived A allele (“Methods”)

As the over-representation of mutations at the RNA editing sites are largely contributed by the sites following an “editing gain model”, we thus focused on these newly originated RNA editing events through G-to-A mutations in the following analyses. On the basis of the macaque polyAG editing sites with an ancestral state of G, we performed site spectrum analysis to investigate the distribution of derived allele frequency of these newly originated RNA editing sites. Considering that the prerequisite of the homozygous A in the macaque animal for A-to-I editing identification may introduce bias of undersampling SNPs with low allele frequency of A, we also introduced a list of non-edited, homozygous A sites with ancestral state of G as a matched control (“Methods”). Notably, the newly originated editing sites have a significantly higher average derived allele frequency than the control (Wilcoxon test, *P* value = 9.33 × 10^− 10^, Fig. 4a). Fay and Wu’s H test [25] further showed a significantly lower Fay and Wu’s H for these newly originated editing sites (*P* value < 1 × 10^− 4^, “Methods”), indicating an excess of high-frequency derived alleles for these sites (Fig. 4a). When further dividing these newly originated editing sites into genic sites (a combination of exonic sites and intronic sites, considering the small number of exonic sites) and intergenic sites, we found that both groups of editing sites showed a significantly higher average derived allele frequency, and a significantly lower Fay and Wu’s H statistic for editing sites than the control sites (Additional file 2: Figure S5), although the selection signals (an excess of high-frequency derived alleles and lower H statistic) are relatively stronger in genic regions than intergenic regions **(**Additional file 2: Figure S5; “Discussion”). These findings thus indicated the general evolutionarily significance for at least a portion of these newly originated editing sites.

As a control, we also perform a similar site spectrum analysis for polyAG sites with ancestral A, and a list of non-edited, homozygous A sites with ancestral state of A was used as a matched control. These editing sites have a slightly lower average derived allele (G) frequency than the control sites (Wilcoxon test, *P* value = 0.01, Fig. 4b), indicating no adaptive signals to maintain these A-to-G mutations which remove the previously existing editing sites.

As a portion of these newly originated RNA editing events are evolutionarily significant, they should have implications in some general biological processes. However, considering the majority of this editome are not connected to well-recognized functional entities, the genome-wide selection signals revealed by population genetics analyses are not readily explicable. The unique subset of primate editome we identified in this study would illuminate the general functions of RNA editing by connecting it to particular gene regulatory processes, based on the characterized outcome of a gene regulatory level in different individuals or primate species with or without these editing events (“Discussion”).

## Discussion

While several case studies have reported that A-to-I editing sites could be genomically encoded as non-editable nucleotides in other species [15, 16], our study is the first genome-wide effort to investigate its generality and underlying mechanism, in human and macaque, especially within the macaque population. The phenomenon was not detected within population in previous studies as the computational pipelines used to identify editing sites were designed to generally remove candidate sites located specifically on previously annotated A/G polymorphic sites [4, 18, 20, 22]. Our findings thus provided a comprehensive atlas (within-population and cross-species) to investigate the recent birth and death of A-to-I editing events through DNA point mutations. In contrast to a recent study by Popitsch et al. [26], we found that the newly originated editing sites following G-to-A mutation contributed predominantly to the over-representation of A/G mutations at editing sites, likely due to the findings that G-to-A mutation site is a favorable location for the origination of robust A-to-I editing event.

The identification and investigation of these newly originated A-to-I editing events also provided a basis for clarifying the evolutionary relevance of RNA editing regulation in primates. Through the population genetics analyses of the focal editing sites, we found that at least a fraction of these young editing events are evolutionarily significant. Besides this approach, the detection of signals for the valley of decreased polymorphism level flanking the editing sites would presumably be additional evidence for the evolutionary significance of the RNA editing events [5, 27]. Accordingly, when comparing the polymorphism levels nearby these newly originated editing sites, on the basis of population genetics data of 31 macaque animals (“Methods”), we could also found a valley of decreased polymorphism level flanking the newly originated macaque hgG editing sites, as compared with the more distal regions as background (Additional file 2: Figure S6). Notably, confounding factors, such as the frequencies of CG dinucleotides, the sequential order between the accumulation of mutations and the introduction of editing, as well as the potential bias introduced in RNA editing identification, may perplex the explanations of the signals. We thus controlled for the CG dinucleotide composition or the nucleotide composition of these regions, the valley of decreased polymorphism level flanking the editing sites could still be detected (Additional file 2: Figure S6, Wilcoxon test, *P* value < 2.2 × 10^− 16^). Especially, to clarify the sequential order between the introduction of editing and the accumulation of mutations, we selected a subset of sites detected as editing in both rhesus macaque and out-group species (green monkey, golden snub-nosed monkey, or black snub-nosed monkey, Additional file 1: Table S1). For these sites, it is plausible according to the parsimony role that the emergence of editing occurred before the divergence between rhesus macaque and crab-eating macaque (Additional file 2: Figure S7A), and the potential bias introduced during RNA editing identification process was also controlled on the branch of crab-eating macaque. We then quantified the mutations accumulated specifically on the branch of crab-eating macaque after the divergence of the two species. Using this list of editing sites with a well-defined sequential relationship between the two events, we still found a valley of a decreased segregation level flanking the ancestral editing sites, as compared with the more distal regions (Additional file 2: Figure S7B, Wilcoxon test, *P* value < 2.2 × 10^− 16^). Overall, although we tried to control for these potential confounding factors, it is plausible that some other confounding factors may still perplex the explanations of the signals; it is thus cautious to use the signals in nearby regions as evidence to support the evolutionary significance of these sites, in contrast to the direct evidence of the focal editing sites.

The newly originated A-to-I editing events are evolutionarily significant in general, indicating that at least a fraction of these sites should already acquire functions during the primate evolution. In order for these RNA editing events to have a significant impact, the editing must occur at sites that could be linked to cellular functions. However, the majority of this editome discussed in this study are located on intronic (60.91%) or intergenic (38.56%) regions, and only a small portion of the identified sites is linked to regions with well-recognized biological functions, such as protein coding (2035 sites or 0.08%). As the majority of this editome are not connected to well-recognized functional entities, the genome-wide selection signals revealed by population genetics analyses are not readily explicable. Therefore, finding new general functions for the pervasive RNA editing sites located in non-coding regions remains a critical issue in the field [1, 11, 12].

Recently, several studies have implicated a portion of RNA editing sites in non-coding regions in other regulatory processes, such as alternative splicing and microRNA regulation [10, 11, 13, 14, 28]. According to a recent study by Liddicoat et al., A-to-I editing of endogenous dsRNA could prevent the activation of the cytosolic dsRNA response by endogenous transcripts, highlighting the functional significance of A-to-I editing events in preventing the MDA5 sensing of endogenous dsRNA. Another study with *ADAR1*-knockdown experiments further linked intronic A-to-I editing to regulations of circRNA biogenesis. We also found that the RNA editing regulation in intergenic regions could crosstalk with piRNA biogenesis and contribute substantially to the diversification of the piRNA repertoire in primates [10, 14, 29]. Besides these studies, several case studies also indicated that inosine-containing RNAs with dsRNA structures could be specifically recognized by paraspeckle and be prevented from exporting to cytoplasm through a NEAT1-based mechanism [30–33]. These findings linking RNA editing regulation to functional entities provide new perspectives on the selection signals maintaining these editing sites. Of note, as a complement to these studies, comparative genomics analyses of the special list of editing sites reported in this study could provide functional connection of RNA editing to these gene regulatory processes: as the editing sites detected in one species/individual were genomically encoded as non-editable nucleotides in the other species/individuals, a cross-species or cross-population comparisons of the outcome of a gene regulatory level may provide clues to the functional implications of these editing sites. Clearly, this is a practical and interesting approach to investigate the general functions of RNA editing in future studies.

## Methods

### Library preparation and deep sequencing

Total RNAs were extracted from frozen tissues following Trizol RNA isolation procedure. The quality of the input RNA was controlled using Agilent 2100. Total RNA samples were then applied to strand-specific, rRNA-depleted RNA-seq, or strand-specific, poly(A)-positive RNA-seq following the pipeline as previously described [10]. Removal of rRNA was performed following the Epicentre Ribo-zero rRNA Removal Kit according to the manufacturer’s protocols. The genomic DNA of macaque brain tissue was extracted using Ultra PureTM Phenol: Chloform: Lsoamyl Alcohol (Invitrogen, 25:24:1, *V*/*V*). Deep sequencing was then performed on Illumina Hiseq X Ten sequencing systems with 151-bp paired-end reads mode.

### Identification and validation of the recent birth and death events of A-to-I RNA editing

The recent birth and death events of RNA editing were identified by first defining A-to-I RNA editing sites detected in some macaque animals while genomically encoded as G in other animals (polyAG, polymorphic editing sites) or in other species (species-specific editing sites). Using a stringent computational pipeline previously reported by us [5, 10], we first identified a list of A-to-I RNA editing sites in six macaque tissues (prefrontal cortex, cerebellum, heart, kidney, muscle, and testis), on the basis of both strand-specific rRNA-depleted RNA-seq and strand-specific poly(A)-positive RNA-seq (Additional file 1: Table S1). Briefly, to address the issue of gapped alignment and control for false positives derived from pseudogene-related misalignments, the RNA-Seq reads were mapped to both the macaque genome (rheMac2) and the transcriptome (Ensembl v85) by BWA (0.7.16a-r1181). A more stringent definition of “uniquely mapped reads” was then used, in that one read was considered to be uniquely mapped only if it had no second-best hit or the second-best hit comprises at least two additional sequence alignment mismatches when considering both the genome and the transcriptome mapping models. Candidate sites with a homozygous genotype were then subjected to additional filtering protocol to remove candidates with low reads coverage, poor base-calling quality, and strand-biased cDNA read distributions. In this pipeline, BWA was selected because it could provide detailed meta-data for short reads mapping (i.e., more comprehensive mapping information of the second-best hits).

To identify the recent birth and death events of RNA editing within the macaque population, the polymorphism map of 31 macaque animals from the RhesusBase PopGateway [23] was used to infer the polymorphism status for each editing site. We further identified the recent birth and death events of RNA editing between human and rhesus macaque. Briefly, the orthologous loci between rhesus macaque (rheMac2) and human (hg19) were identified using LiftOver with default parameters. Genotyping data from the 1000 Genomes Project (Phase 3) [34] were further used to clarify whether the macaque-specific A-to-I editing events are genomically fixed as G in the human population. As for the background sequence nearby the focal editing sites, only sites with homozygous adenines in the macaque animal were used, which is a requirement similarly applied to the identification of RNA editing sites. To experimentally verify some of these events, candidate sites with editing level ≥ 10% were selected randomly, as a threshold for the detection sensitivity of Sanger sequencing. DNA isolated from whole blood samples of six rhesus macaque animals (TIANGEN, DP304–03), as well as from saliva of six healthy human individuals (CWBIO, CW2611), were then used in PCR amplification and Sanger sequencing.

### Targeted DNA sequencing

For 54 randomly selected polyAG sites (Additional file 4: Table S4), we performed targeted capturing by using probes designed by SureSelect System (Agilent Technologies, Inc., Santa Clara, USA) in a population of 82 unrelated male macaque animals [35]. Briefly, DNA oligo probes were designed by Target Enrichment System to capture the targeted regions. As most of the editing sites were located on highly repetitive *Alu* elements, probes covering the editing sites might not reach the desired specificity. We thus designed the probes corresponding to the nearby non-repetitive regions at a distance of about 100 bp to the focal editing sites. Three micrograms genomic DNA was then isolated from whole blood sample of each macaque animal, using the QIAamp DNA Blood Mini Kit (Qiagen, Venlo, Netherlands). The genomic DNA was then sheared to fragments with a peak at 150–200 bp for the library construction following Agilent SureSelect XT Target Enrichment System’s instructions. Deep sequencing was performed on Illumina Miseq sequencing system, with 151 bp paired-end reads mode. After reads mapping to macaque genome (rheMac2), single nucleotide polymorphisms were then identified according to the standard GATK pipeline with HaplotypeCaller.

### Ancestral allele definition and population genetics analyses

The ancestral state of macaque editing site was inferred following the Enredo-Pecan-Ortheus (EPO) pipeline [36]. The proportions of sequence differences between human and macaque, as well as the proportions of polymorphic sites within a macaque population of 31 animals [23] were calculated with in-house scripts, for species-specific editing sites and polymorphic editing sites, respectively. As a genomic background, the adjacent homozygous non-edited A sites for each editable A (within 25 bp upstream or downstream the focal editing sites) were combined to calculate these proportions.

For the newly originated polymorphic editing sites in rhesus macaque with ancestral state of G, the derived allele was defined by the EPO pipeline from eight primate species (human, gorilla, chimpanzee, orangutan, macaque, African green monkey, baboon, and marmoset), in which the ancestral state for macaque and baboon was used to polarize the polymorphism site. In this pipeline, the potential mutation bias of CpG regions was considered in steps to simulate local sequence-dependent fluctuations in substitution and indel rates [36]. A site frequency spectrum for the derived A allele was then estimated with 10,000 times of bootstrap to deduce the confidence intervals. As the prerequisite of the homozygous A in the macaque animal for A-to-I editing identification may introduce bias of undersampling SNPs with low allele frequency of A, we introduced a list of non-edited, homozygous A sites with ancestral state of G as a matched control. The Wilcoxon tests were performed to compare the average derived allele frequency between the two datasets. Fay and Wu’s H test [25] was also performed to investigate whether an excess of high-frequency derived alleles exists for newly originated editing sites. The significance of this test was further determined by comparing the H score of newly originated editing sites against the distribution generated by 10,000 times of bootstrap of the control sites. Similarly, a site frequency spectrum for the derived G allele for polyAG-ancestral A sites was then estimated and compared with matched control of non-edited, polyAG sites with ancestral state of A. The Wilcoxon tests were performed to compare the average derived allele frequency between the two datasets.

On the basis of the polymorphism data from the population of 31 macaque animals, we also estimated the polymorphism levels (π) for the nearby regions and remote regions of A-to-I RNA editing sites. The distribution of nucleotide diversity nearby these editing sites was also calculated as a genomic background with 10,000 times of bootstrap to deduce the confidence intervals.

### RNA secondary structure prediction

For RNA editing sites within *Alu* elements, the nearest inverted *Alu* pairs were located. The genomic distance between two inverted *Alu*s should be less than 5000 nt and on the same transcript. The RNA secondary structure was then predicted by MFOLD (v3.6) [37].

## Additional files

Additional file 1: **Table S1.** Statistics of the deep sequencing data used in this study. (XLSX 12 kb)

Additional file 2: **Table S2.** Genomic annotation of the A-to-I RNA editing sites in *Alu* elements. Table S5. Proportion of transcribed, *Alu*-associated hgG or polyAG sites with different ancestral status detected as editing. Figure S1. Accurate editome identification in rhesus macaque. Figure S2. Over-representation of A/G divergent and polymorphic sites at RNA editing sites. Figure S3. Proportions of human-macaque sequence differences and macaque polymorphic sites. Figure S4. Distribution of editing levels for various editing type in different tissues. Figure S5. Site frequency spectrum for derived A allele for polyAG editing sites with ancestral G. Figure S6. The polymorphism levels of newly originated RNA editing events compared with remote regions. Figure S7. Quantification of mutations accumulated after the origination of editing events. (DOCX 4790 kb)

Additional file 3: **Table S3.** A-to-I editing events located on polymorphic sites in the macaque population. (XLSX 1879 kb)

Additional file 4: **Table S4. 54** randomly selected polyAG sites of targeted DNA sequencing. (XLSX 13 kb)
