# Supplementary material for: Evolutionarily significant A-to-I RNA editing events originated through G-to-A mutations in primates
Source: Genome Biol. 2019 Feb 4;20:24. doi: 10.1186/s13059-019-1638-y (PMC6360793; doi:10.1186/s13059-019-1638-y)
Supplement: Supplementary file 2 — Table S2. Genomic annotation of the A-to-I RNA editing sites in Alu elements. Table S5. Proportion of transcribed, Alu-associated hgG or polyAG sites with different ancestral status detected as editing. Figure S1. Accurate editome identification in rhesus macaque. Figure S2. Over-representation of A/G divergent and polymorphic sites at RNA editing sites. Figure S3. Proportions of human-macaque sequence differences and macaque polymorphic sites. Figure S4. Distribution of editing levels for various editing type in different tissues. Figure S5. Site frequency spectrum for derived A allele for polyAG editing sites with ancestral G. Figure S6. The polymorphism levels of newly originated RNA editing events compared with remote regions. Figure S7. Quantification of mutations accumulated after the origination of editing events. (DOCX 4790 kb) [file 13059_2019_1638_MOESM2_ESM.docx]

**Additional file 2**

**Table S2.** **Genomic annotation of the A-to-I RNA editing sites in *Alu* elements.**

| **Region** | ***Alu*-specific** |  |
| --- | --- | --- |
| Intronic | 1,607,384 |  |
| Intergenic | 1,017,542 |  |
| 3’ UTR | 11,290 |  |
| CDS | 2,035 |  |
| 5’ UTR | 587 |  |

**Table S5. Proportion of transcribed, *Alu*-associated hgG or polyAG sites with different ancestral status detected as editing.**

| Type | Proportion of sites detected as edited |
| --- | --- |
| hgG-ancG | 6.84% |
| hgG-ancG Control | 6.05% |
| hgG-ancA | 5.01% |
| hgG-ancA Control | 5.58% |
| polyAG-ancG | 6.82% |
| polyAG-ancG Control | 6.18% |
| polyAG-ancA | 3.76% |
| polyAG-ancA Control | 5.65% |

*The transcribed, *Alu*-associated hgG or polyAG sites were divided into different categories according to their ancestral status. For each category of sites, matched controls were introduced, for which the variation of position-dependent mutation rates were controlled in the same trinucleotide context.


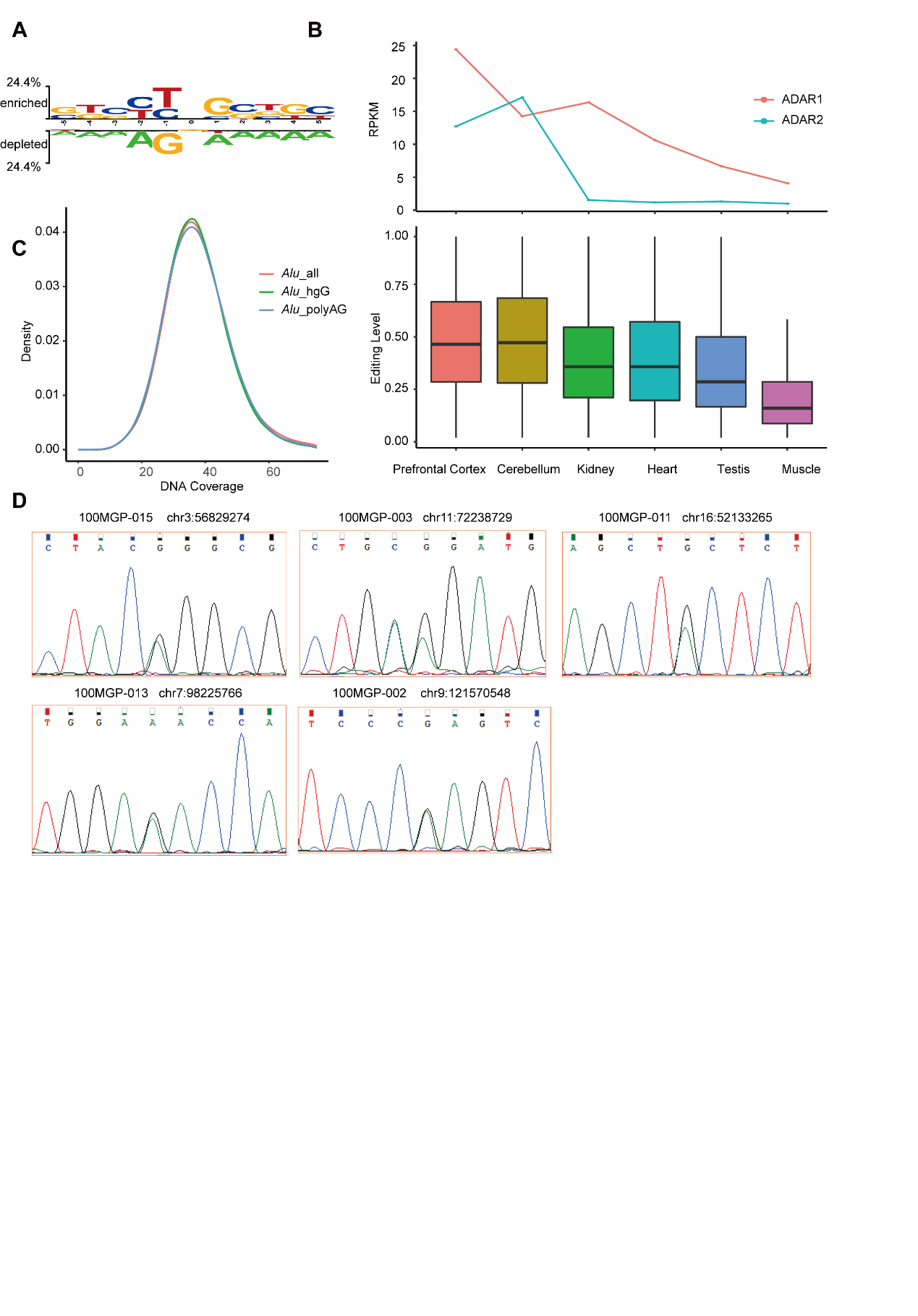


**Figure S1. Accurate editome identification in rhesus macaque. (A)** The enriched (above the top line) and depleted (below the bottom line) nucleotides nearby the focal *Alu*-specific editing sites are displayed in Two-Sample Logo, with the level of preference/depletion shown in height proportional to the scale. **(B)** The tissue expression profiles of *ADAR1* or *ADAR2* were ordered based on ribo-minus RNA sequencing data (**upper panel**). *Alu*-specific A-to-I RNA editing levels for six tissues are shown in the same order as in **(lower panel)**. **(C)** The coverage distribution of DNA sequencing for the full list of editing sites (***Alu*_All**), polyAG editing sites (***Alu*_polyAG**) and hgG editing sites (***Alu*_hgG**) located on *Alu* regions. **(D)** Sanger sequencing validation for **polyAG** RNA editing sites which are A/G polymorphic in the macaque population.

**
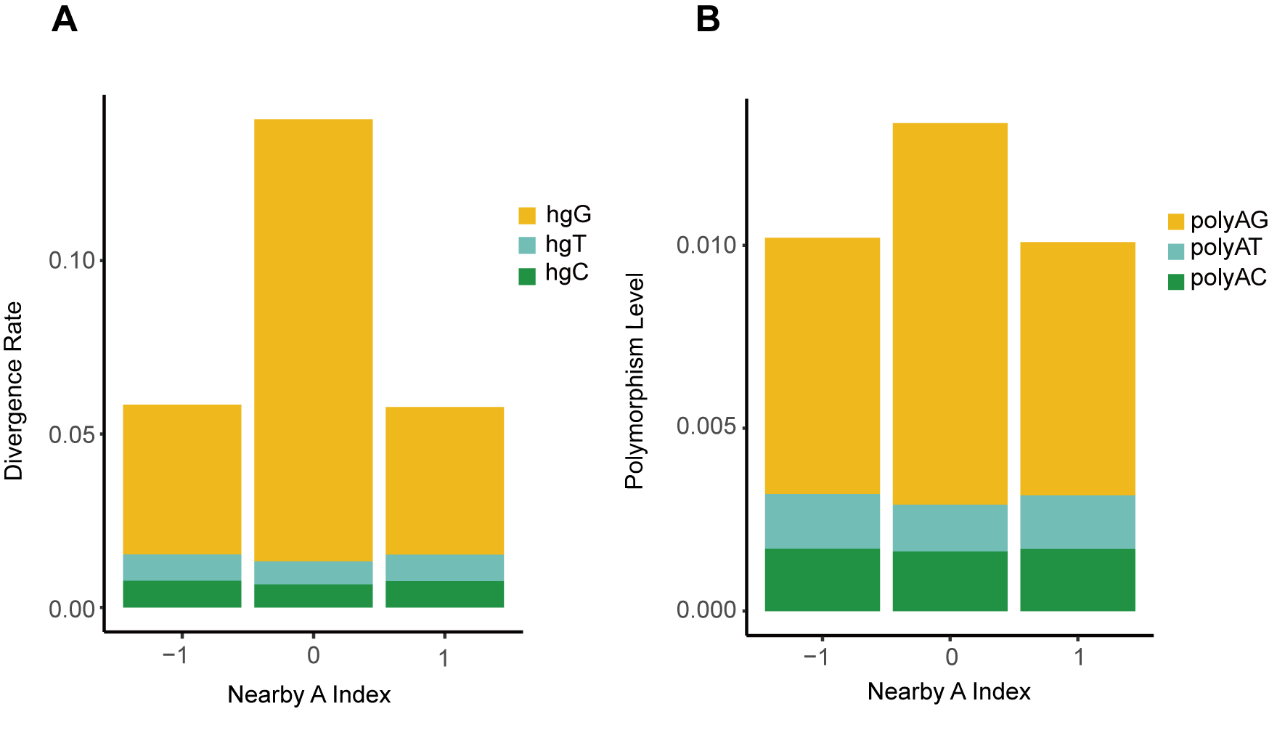
**

**Figure S2. Over-representation of A/G divergent and polymorphic sites at RNA editing sites. (A, B)** The distributions of human-macaque sequence divergence rate **(A)** and polymorphism level **(B)**, controlled for the variation of position-dependent mutation rates, are shown for the focal A-to-I RNA editing sites and the adjacent homozygous A sites.


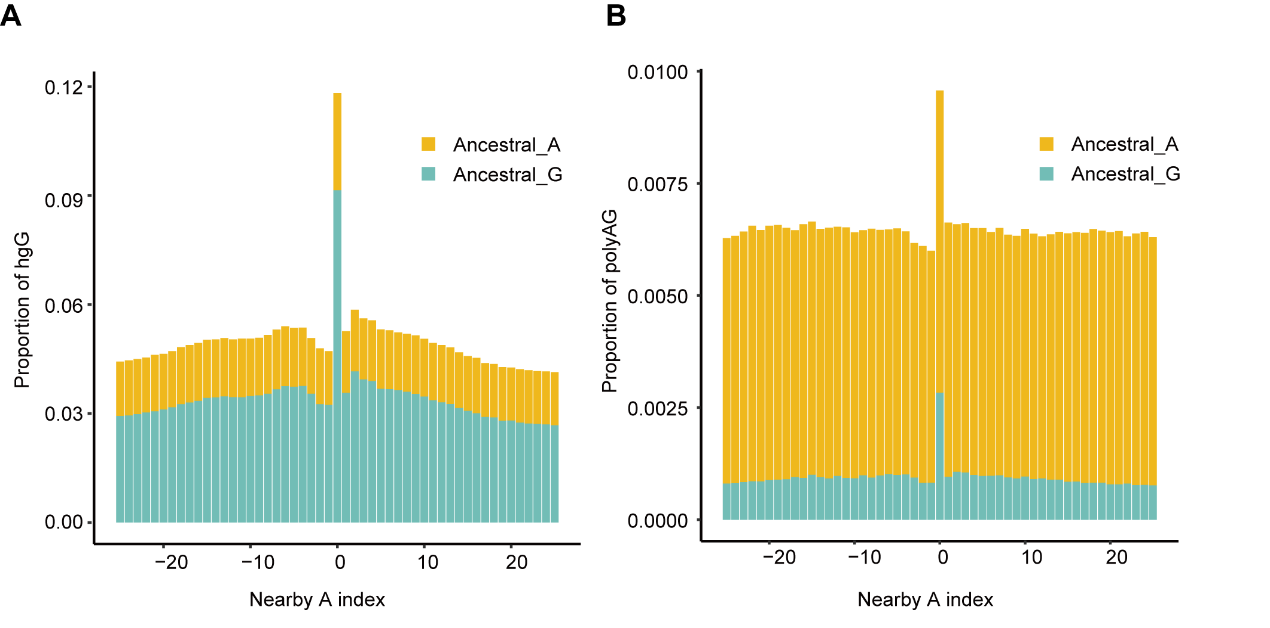


**Figure S3. Proportions of human-macaque sequence differences and macaque polymorphic sites. (A)** The distribution of the proportions of human-macaque sequence differences is shown for the focal RNA editing sites (**Index 0**) as well as the nearby non-edited, homozygous A sites within 25bp upstream or downstream of the focal editing sites (**Nearby-A Index**). (**B**) The distribution of the proportions of polymorphic sites within macaque population is shown for the focal editing sites (**Index 0**) and the nearby homozygous A sites within 25bp upstream or downstream of the focal editing sites (**Nearby-A Index**). The sites were classified into two groups according to the ancestral status.


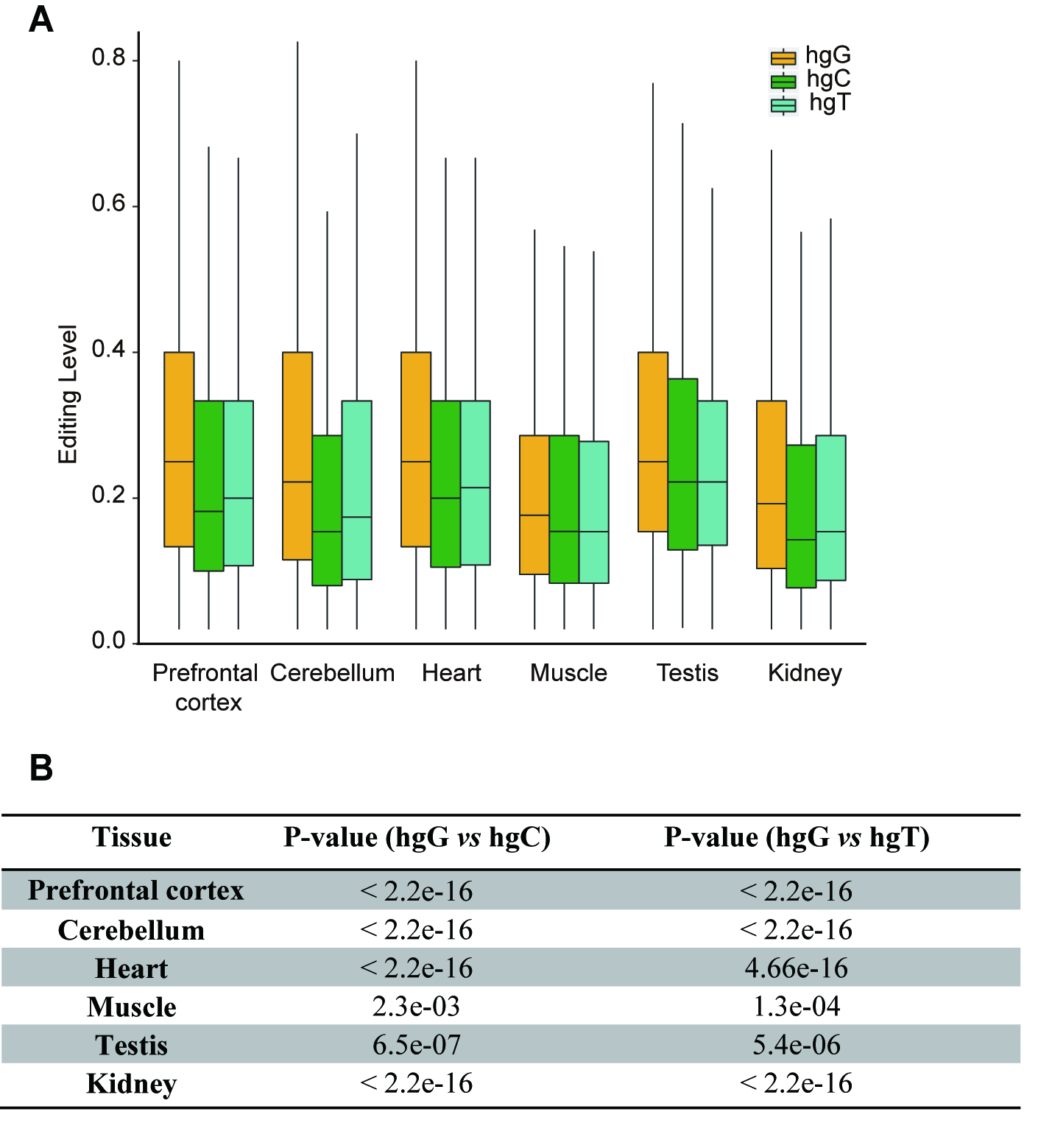


**Figure S4. Distribution of editing levels for various editing type in different tissues.** The editing levels in six tissues are calculated and shown respectively for **hgG**, **hgC** and **hgT** groups of editing sites **(A)**, with the *P*-values of Wilcoxon tests shown (**B**).


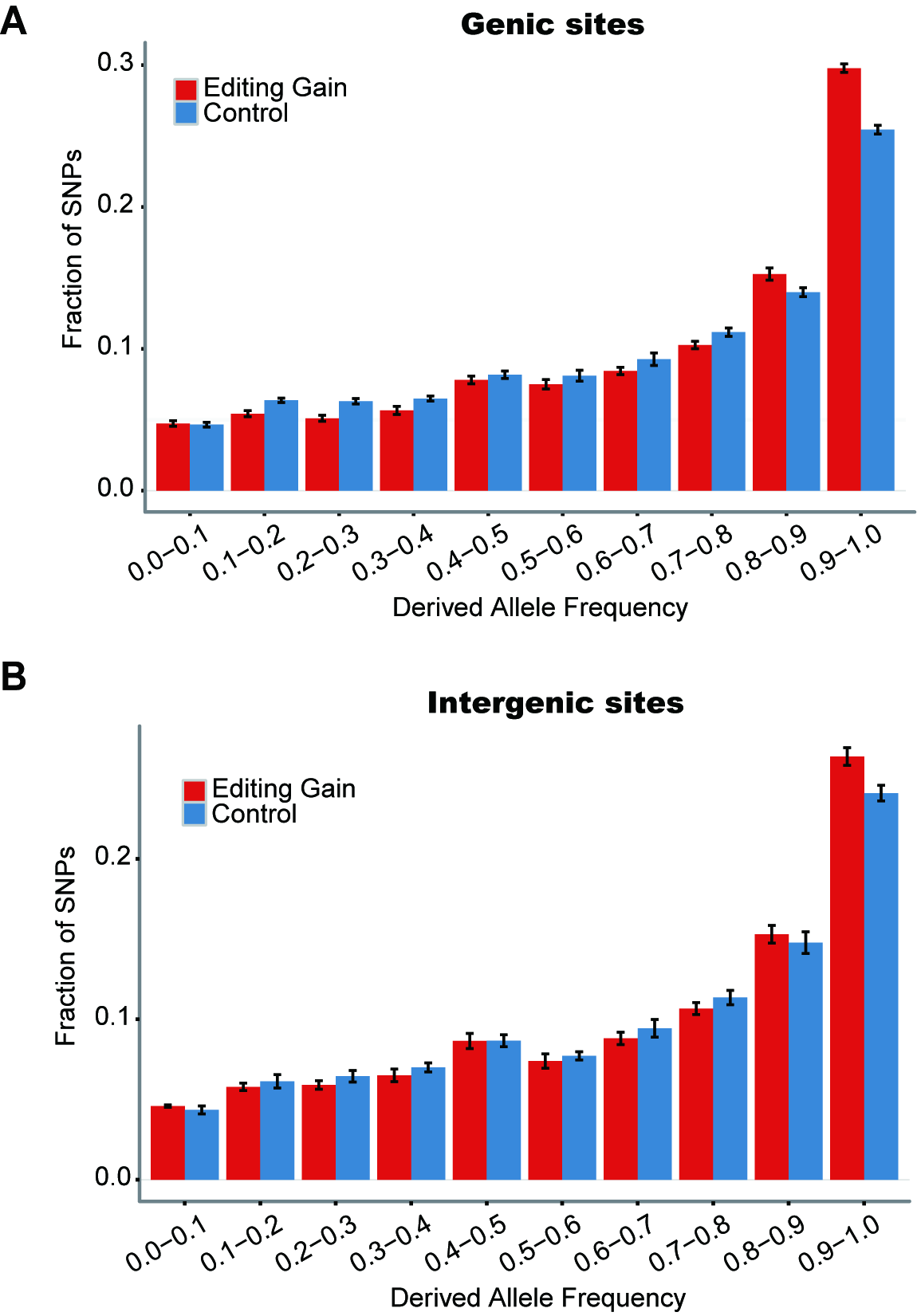


**Figure S5. Site frequency spectrum for derived A allele for polyAG editing sites with ancestral G.** For the polyAG editing sites with ancestral state of G, a site frequency spectrum for the derived A allele is shown (**Editing Gain**). As a background, a list of non-editing, homozygous A sites with ancestral state of G (**Control**) were also used to generate a site frequency spectrum for derived A allele (**Methods**). The distributions of site frequency spectrum are shown for editing sites located in genic (**A,** H score=-6.77, *P*-value<1×10^-4^, 10,000 times bootstrap of control sites) and intergenic regions (**B,** H score=-6.27, *P*-value=1.51×10^-2^), respectively.


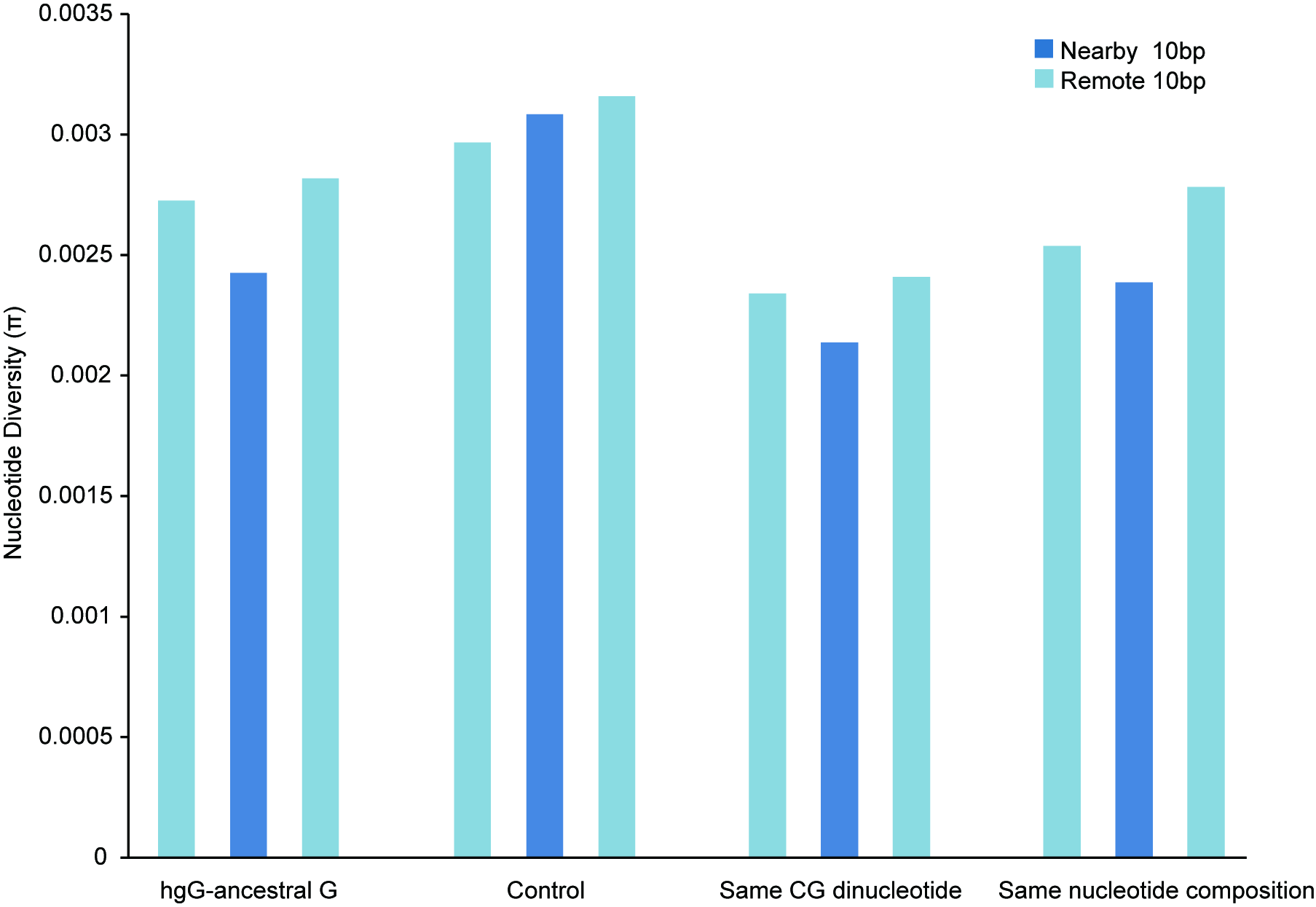


**Figure S6. The polymorphism levels of newly-originated RNA editing events compared with remote regions.** The nucleotide diversity (π) nearby the focal sites were calculated and shown in the nearby 10 bp-window and two remote 10 bp-windows (**Methods**) for four groups of sites. **hgG-ancestral G**: macaque hgG editing sites with ancestral state of G; **Control**: the non-edited, homozygous hgG A sites with ancestral state of G; **Same CG dinucleotide:** editing sites with the same percentage of CG dinucleotide in the nearby and remote regions; **Same nucleotide composition**: editing sites with the same nucleotide composition in the nearby and remote regions.


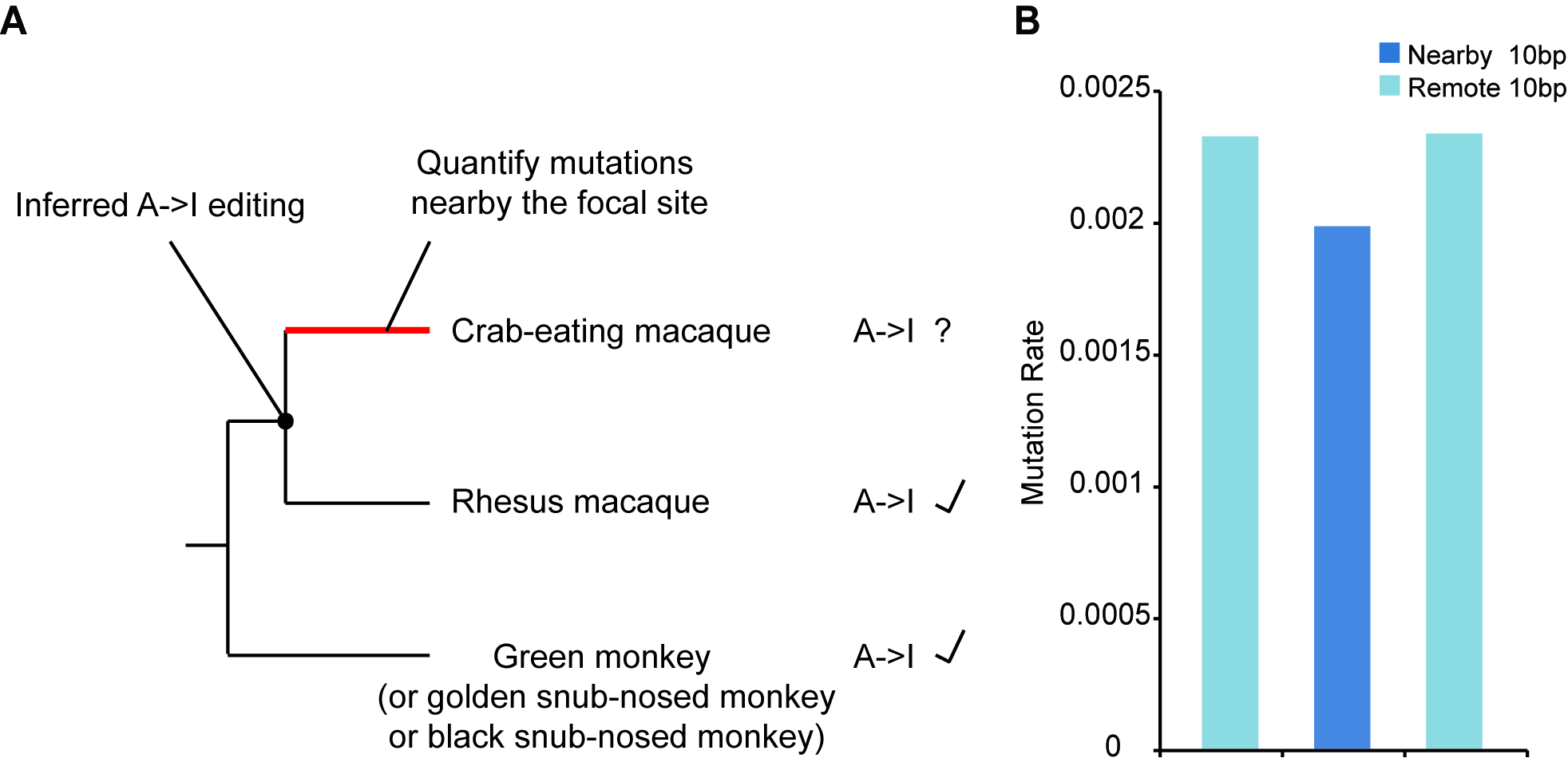


**Figure S7. Quantification of mutations accumulated after the origination of editing events**. **(A)** Diagram shown the principles in identifying the recent mutations accumulated specifically in the branch of crab-eating macaque after the origination of editing events at the common ancestral of crab-eating macaque and rhesus macaque. The rates of the mutations accumulated specifically in the branch of crab-eating were calculated and shown in **(B),** for the nearby 10 bp window flanking the ancestral editing sites, and two remote 10bp windows.
